# Acupuncture for patients with atopic dermatitis: A systematic review protocol

**DOI:** 10.1097/MD.0000000000018559

**Published:** 2019-12-27

**Authors:** Yijiang Liu, Hai Cui, Ruosang Du, Lu Zhang, Hongwen Yuan, Xiaohui Zhang, Shumei Zheng

**Affiliations:** School of Traditional Chinese Medicine, Capital Medical University, Beijing, China.

**Keywords:** acupuncture, atopic dermatitis, protocol, systematic review

## Abstract

**Background:**

The systematic review protocol is aim to evaluate the efficacy and safety of acupuncture in the treatment of atopic dermatitis (AD).

**Methods:**

We will search the database on the Cochrane Library, PubMed, Medline, Excerpta Medica Database, Chinese Biomedical Literature Database, PsychINFO, China National Knowledge Infrastructure, Wanfang data, Chinese Scientific Journal Database, including studies and published systematic review in the reference list and grey. And will use Cochrane Collaboration's tools to evaluate the risk of bias of the included randomized controlled trials. The Review Manager 5.3 will be used to synthesize collected data.

**Results:**

This study will assess the safety and effectiveness based on current evidence of acupuncture for AD, especially scoring atopic dermatitis, eczema area, and severity index, patient-oriented eczema measure, and quality of life.

**Conclusion:**

This study will provide high-quality recently evidence for evaluating the efficacy of acupuncture for patients with AD.

PROSPERO registration number: CRD42019135919.

## 1 Introduction

Atopic dermatitis (AD) is a complex chronic inflammatory disease of the skin, which is closely related to other atopic and allergic diseases, including asthma and atopic rhinitis.[^1^,^2^,^3^] It is characterized by recurrent skin inflammation and dysfunction of the epidermal barrier.^[4]^ Its etiology is not clear, but it is related to genetic susceptibility, food, and inhalation allergen stimulation, auto-antigen, infection, and skin dysfunction. The prevalence of AD in children is about 15% to 20% and in adults is 1% to 3%. The incidence of AD in industrialized countries has increased by 2 to 3 times in the past few decades.^[5]^

The symptoms of AD include red or brown skin plaque, dry, cracked or scaly skin, and itching. It often manifests as persistent itching throughout the day, which can aggravate and lead to insomnia at night, affecting the quality of life of patients,^[2]^ so itching management is very important. At present, mainstream treatment is to prevent the aggravation of the disease and reduce skin inflammation. Moisturizers, local steroids, calcineurin inhibitors, and H1 antihistamines are selected. Long-term local use of corticosteroids can cause local and systemic adverse reactions.^[6]^

Because of the limited medical effect of AD, herbal medicine, and acupuncture are more used in AD,[^7^,^8^,^9^,^10^,^11^,^12^] as complementary and alternative therapies. Acupuncture has been widely used in China for thousands of years and previous studies have shown that compared with placebo and sham acupuncture groups, both acupuncture and cetirizine can significantly reduce pruritus symptoms in AD patients.^[13]^ In Quchi (LI11) acupoint pressing for 4 weeks, subjects improved pruritus, lichenization, and quantitative assessment of itch intensity (visual analog scale [VAS] ) decreased significantly.^[14]^

The previous searching of the electronic database, relevant randomized clinical controlled trials were updated. Due to the limitations of clinical basis and practice, previous systematic reviews considered that no qualified research could be included, and no conclusion was reached.^[15]^ Therefore, it is necessary to make a meta-analysis of AD to evaluate the effectiveness and safety of acupuncture in the treatment process. In this systematic review, we will evaluate the efficacy and safety of acupuncture for treating AD.

## 2 Methods

The protocol was registered on Prospero (CRD42019135919). The content followed preferred reporting items for systematic review and meta-analysis protocols (PRISMA-P).^[16]^

### 2.1 Eligibility criteria

#### 2.1.1 Types of studies

This review will include all randomized controlled trials (RCTs) in English or Chinese and without the restriction of publication type. Non-RCTs, quasi-RCTs, uncontrolled trials, case series, case reports, crossover studies, letters, and laboratory studies will be excluded.

#### 2.1.2 Types of participants

Patients with AD were diagnosed according to Hanifin and Rajka criteria or the UK refinement were included.[^17^,^18^,^19^] Information relating to their age, sex, race, education, nationality, or economic status is not taken into account.

#### 2.1.3 Types of interventions

##### 2.1.3.1 Experimental interventions

We will include the acupuncture therapies that are reported in studies (body acupuncture, electroacupuncture, intradermal needle, auricular acupuncture, scalp acupuncture, ocular acupuncture, fire needling, elongated needling, and plum blossom needle), exclude acupoint injection, laser acupuncture, moxibustion, cupping, and acupressure.

##### 2.1.3.2 Control interventions

Comparisons interventions include placebo control, sham acupuncture, drug therapy, other therapies, no therapy, and acupuncture plus 1 treatment compares with the same treatment. Besides, the following comparisons of studies will be excluded:

(1) Comparing in the acupuncture plus different therapies;
(2) Comparing in different acupuncture therapies;
(3) Comparing acupuncture plus a therapy with another therapy.

#### 2.1.4 Types of outcome measures

##### 2.1.4.1 Primary outcomes

The primary outcomes included clinical disease severity measured by 1 or more of the following instruments,

(1) Scoring atopic dermatitis[^19^,^20^];
(2) Eczema area and severity index [^20^,^21^];
(3) Patient-oriented eczema measure [^20^,^22^];
(4) VAS (pruritus, insomnia)^[23]^;

##### 2.1.4.2 Secondary outcomes

The secondary outcomes included,

(1) Changing in patients’ quality of life (ie, dermatology life quality index; children's dermatology life quality index; infants’ dermatology life quality index)[^24^,^25^,^26^];
(2) The recurrence rate;
(3) Side effect and adverse events;

### 2.2 Search methods for the identification of studies

The following databases will be searched: The Cochrane Library, PubMed, Medline, Excerpta Medica Database, PsychINFO, Chinese Biomedical Literature Database, China National Knowledge Infrastructure (CNKI), Wanfang database, Chinese Scientific Journal Database. In addition, relevant references and RCTs cited in the reports will also be searched as supplementary sources. We will also search the gray literature, for example, conference proceedings and unpublished study in CNKI and Wanfang.

Relevant keywords were used to create search strategies which in PubMed were listed in Table 1. In the selection process, only research conducted in humans will be included in further review. The PubMed search strategy in Table 1 will be adapted for other databases.

**Table 1:**
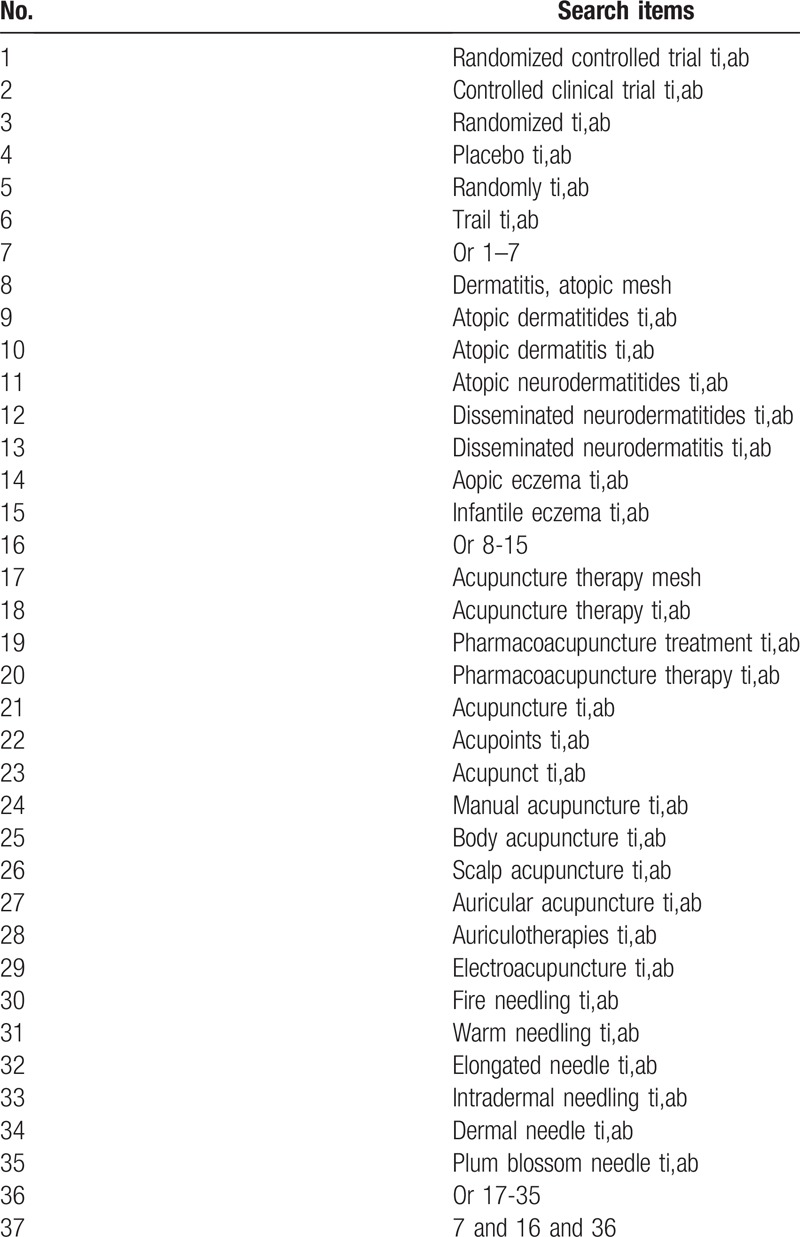
Search strategy used in PubMed database.

### 2.3 Data collection and analysis

#### 2.3.1 Selection of studies

**Figure 1:**
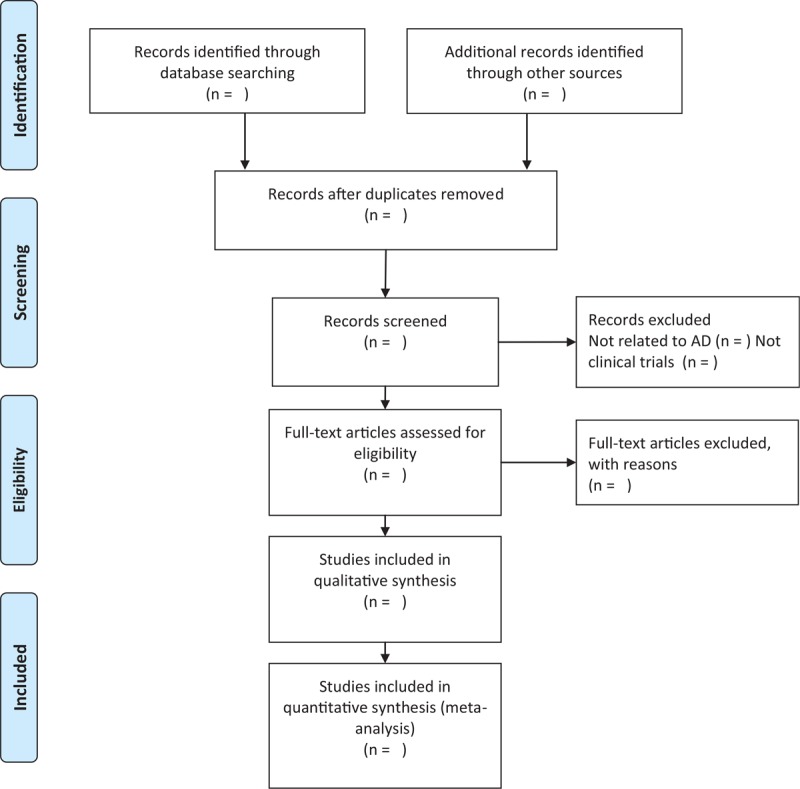
PRISMA flow diagram of study process. PRISMA = preferred reporting items for systematic reviews and meta-analysis.

Three reviewers (YJL, HC, and RSD), who will be told the aim and process of the system review, will select the trials and studies independently according to the criteria for inclusion by reading the titles and abstracts and if necessary, the full text will be read for further assessment. The discrepancies in the process will be discussed and solved with SMZ. Details of the research choices are shown and exclusive studies will be listed and explained. The process of study selection will be presented in PRISMA flow diagram (Fig. 1).

To ensure consistency, we will perform calibration exercises on methodological steps of the review process before assessment.

#### 2.3.2 Data extraction and management

Three reviewers (YJL, HC, and RSD) will separately extract the data from the study into the form which is written by the Cochrane skin group and we have modified it to accommodate this review. We will extract information such as author, time of publication, participants information (age, sex, disease duration), interventions, outcome indicators, adverse events, and other details. If a disagreement of 3 reviewers cannot be resolved when extracting data, it will be solved by consulting and discussing with SMZ. Any discrepancies in the process will be discussed and solved with SMZ.

#### 2.3.3 Risk of bias assessment

Cochrane risk of bias assessment tools will be used to evaluate 6 domains of bias (sequence generation, allocation concealment, blinding, incomplete outcome data, selective outcome reporting, and other sources of bias).^[27]^ The included studies will be divided into 3 parts: unclear, low risk, and high risk. Any discrepancies in the process will be discussed and solved with SMZ.

#### 2.3.4 Assessment of heterogeneity

*I* square (*I* ^*2*^) statistic will be used to assess heterogeneity in included studies, which describes the percentage of variation between studies due to heterogeneity. When *I* ^*2*^ < 50%, the fixed-effect model will be selected to calculate risk ratio (RR) and mean difference (MD), otherwise use a random-effect model.

#### 2.3.5 Measures of treatment effect

We used review manager 5.3 for meta-analysis to synthesize collected data. For continuous variables, we will use MD or standardized mean difference with 95% confidence interval (CI). For dichotomous data will be calculated the RR with 95% CI.

#### 2.3.6 Dealing with missing data

If including studies data is insufficient, we will contact the original authors for relevant information. The intent-to-treat analysis will be applied to include studies if the missing data cannot be found.

#### 2.3.7 Subgroup analysis and investigation of heterogeneity

If data are available, we will do a subgroup analysis base on the types of acupuncture intervention and different progressions of AD will also be analyzed. The impact of method quality, sample size, missing data, and analysis methods will also be evaluated by sensitivity analysis.

#### 2.3.8 Ethics and dissemination

The system evaluation does not require ethical approval, because it is not individual data that is used. The results will provide evidence for the efficacy and safety of acupuncture in the treatment of AD. They will also have an impact on clinical practice and research.

#### 2.3.9 Assessment of reporting biases

We will use a funnel plot to test reporting biases if more than 10 trials are included in the meta-analysis.

## 3 Discussion

AD is a disease with limited clinical treatment. There is no definite conclusion although several studies have reported that acupuncture can help AD patients ease symptoms. Therefore, this study will discuss the effectiveness and safety of acupuncture for patients with AD, and provide a systematic and comprehensive evaluation for the future practice of AD treatment.

The review may have some limitations which are deserved attention. We only include English and Chinese studies, and other language studies may be missed and different forms of acupuncture treatments, acupoint selection, disease severity, and quality of methodology may cause significant heterogeneity.

## Author contributions

SMZ is the lead and the guarantor of this review. YJL, HC, and RSD conceptualized the review and drafted the manuscript. HWY, LZ, and XHZ set up the search strategy included in the protocol. YJL and HC revised the protocol critically. All authors read and provided amendment on the draft and confirmed the final manuscript.

Yijiang Liu orcid: 0000-0001-5971-6175.
